# Bedaquiline and delamanid in the treatment of multidrug‐resistant tuberculosis: Promising but challenging

**DOI:** 10.1002/ddr.21498

**Published:** 2018-12-11

**Authors:** Yang Li, Feng Sun, Wenhong Zhang

**Affiliations:** ^1^ Department of Infectious Diseases Huashan Hospital, Fudan University Shanghai China; ^2^ State Key Laboratory of Genetic Engineering School of Life Science, Fudan University Shanghai China

**Keywords:** bedaquiline, delamanid, multidrug‐resistant tuberculosis

## Abstract

Improving treatment outcomes in multidrug‐resistant tuberculosis (MDR‐TB) is partly hampered by inadequate effective antitubercular agents. Development of bedaquiline and delamanid has potentially changed the treatment landscape for MDR‐TB. This review provides an update on the progress of these novel antitubercular agents. We review published studies aimed at evaluating clinical efficacy and effectiveness of bedaquiline and delamanid. Five prospective clinical studies and seven retrospective studies on bedaquiline showed that patients treated with a bedaquiline‐containing regimen had a high culture conversion rate ranging from 65 to 100% and a satisfactory treatment outcome. The combined use with linezolid might add to the effectiveness of bedaquiline. Controversies about bedaquiline resistance are discussed. Three clinical trials have reported outcomes on delamanid and showed that introducing delamanid to a background regimen improved culture conversion rate at 2 months from 29.6% to more than 40%. A higher favorable treatment rate was also observed among patients who received delamanid for more than 6 months, but about a quarter of patients defaulted in the control group. Seven retrospective studies were summarized and found a treatment benefit as well. More reliable evidence from randomized clinical trials reporting on the treatment outcomes is needed urgently to support a strong recommendation for the use of delamanid. Advances in the combined use of bedaquiline and delamanid are also reviewed, and the combination may be well tolerated but requires electrocardiograph monitoring.

## 1 INTRODUCTION

*Mycobacterium tuberculosis* has developed the ability to continually resist the antitubercular agents despite the use of potent drugs in various combinations to constitute treatment regimens. Drug‐resistant tuberculosis (TB) is emerging at an alarming rate throughout the world, with 460,000 patients contracting with multidrug‐resistant tuberculosis (MDR‐TB) in 2017 (World Health Organization, WHO, 2018a). MDR‐TB is defined as TB resistant to both rifampicin and isoniazid. In addition, extensively drug‐resistant TB (XDR‐TB), defined as MDR with additional resistance to any fluoroquinolone and to second‐line injectable agents, represented 8.5% of all patients with MDR‐TB, and this has been posing a serious threat to the global TB control (WHO, 2018b). Here, we used the terms simple MDR‐TB to refer to TB that is resistant to just rifampicin and isoniazid but not to the fluoroquinolones and/or the second‐line injectable agents, whereas complicated MDR‐TB refers to additional resistance to either (pre‐XDR) or both of these drug groups (XDR‐TB) (Zhao et al., 2009).

It is imperative to exploit new drugs for the treatment of drug‐resistant TB. Among a dozen of new antitubercular agents, bedaquiline (Janssen, Beerse, Belgium) and delamanid (Otsuka, Tokyo, Japan) are the most frequently investigated drugs (Tiberi et al., 2018), and the early promising results of these two drugs inspired clinicians with confidence. Inevitably, the confidence of better treatment outcomes is paired with the concerns about the acquired resistance to new drugs (Bloemberg et al., 2015; Veziris et al., 2017). In addition, unlike drug‐susceptible TB, failure to convert to negative culture remains the most problematic aspect in treating patients with MDR‐TB (Cegielski et al., 2014). Here, we aimed to describe to what extent bedaquiline and delamanid can improve favorable treatment outcomes for individuals with MDR‐TB and the challenge facing clinicians on how to better use these drugs. Moreover, the drugs' effect on culture conversion should be assessed and discussed as a priority.

In June 2018, we used PubMed database to search the articles written in English published up to June 1, 2018. We selected the articles reporting on the efficacy, effectiveness of any regimens containing bedaquiline and/or delamanid in adult patients (>18 years of age) with MDR‐TB. Articles were included in the search if they contained “Bedaquiline” or “TMC207” or “Delamanid” or “OPC‐67683” in the title, and two authors further evaluated all papers' titles and abstracts. Original articles and letters in this scope were selected. Case reports, reviews, and editorials are not described here.

A total of 266 articles were identified. Among them, 208 articles were removed after reviewing the titles and abstracts, including 39 reviews, 10 case reports, 4 errata, 4 editorials, 7 guidance or instruction, 2 articles targeting children and pregnant woman, 13 articles focusing on nontuberculous mycobacteria infections, 54 articles concerning basic medicine researches or model‐based analyses, 28 articles reporting drug resistance mechanisms or drug‐susceptibility testing methods, 33 articles studying the pharmacokinetics and pharmacodynamics of the drugs, and 14 articles reporting the drugs' access and tolerability. After the full review, 30 comments or viewpoints were removed because of the lack of original clinical data. Finally, a total of 28 publications were included in this review.

### 1.1 Bedaquiline

Bedaquiline deserves particular attention because it is the first antitubercular agent approved by the U.S. Food and Drug Administration (FDA) since the late 1970s (Osborne, 2013). By March 2018, approximately 16,639 patients had received bedaquiline. Bedaquiline's potent bactericidal and sterilizing activity (Caminero, Piubello, Scardigli, & Migliori, 2017; Diacon, Dawson, et al., 2012) are accredited to its unique and specific mechanism as a mycobacterial ATP synthase proton pump inhibitor (Andries et al., 2005) and its effect on further remodeling bacterial metabolism (Koul et al., 2014). Combining other bactericidal drugs such as third‐generation fluoroquinolones, pretomanid (PA‐824), or pyrazinamide with bedaquiline significantly increased and advanced early bactericidal activity (EBA) compared to bedaquiline alone (Diacon, Dawson, et al., 2012). The terminal elimination half‐life of bedaquiline is discernibly longer than that of the other antitubercular agents, up to 5–6 months (McLeay, Vis, Van Heeswijk, & Green, 2014), and this pharmacokinetic characteristic tends to greatly affect the duration of bedaquiline administration in clinical application.

#### 1.1.1 Promising results of bedaquiline in improving the sputum culture conversion rate and treatment outcomes

**Figure 1:**
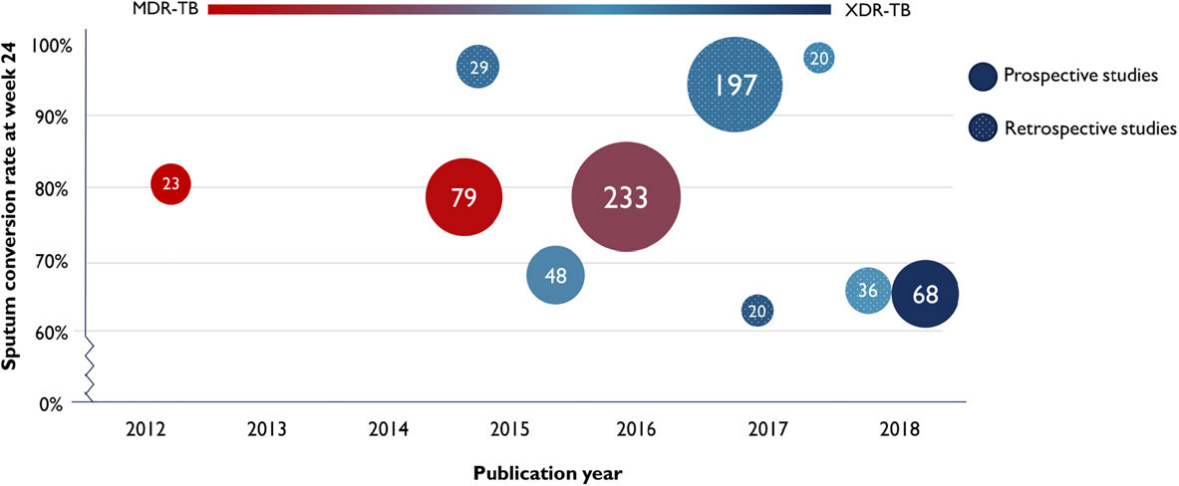
Summary of prospective and retrospective studies reporting on the sputum conversion rate at week 24 among patients treated with the bedaquiline‐containing regimen. Each circle represents one clinical study, and spotted circles were retrospective studies. The number in the middle of circle is the sample size enrolled into studies. The shade of color represents the proportion of patients with XDR‐TB in the study, like the circle in indigo blue means that the corresponding study only enrolled patients with XDR‐TB and the circle in red means that all participants were simple MDR‐TB. MDR‐TB = multidrug‐resistant tuberculosis; XDR‐TB = extensively drug‐resistant tuberculosis

As of June 1, 2018, six articles had been published to report prospective clinical studies on bedaquiline (Figure 1, Table 1). Studies on the combination of bedaquiline and delamanid will be discussed later in this article. The three earliest articles (Diacon et al., 2009; Diacon, Donald, et al., 2012; Diacon et al., 2014) presented the results of Trial C208. Trial C208, a phase 2b, randomized controlled trial conducted by Diacon et al. was launched among newly diagnosed patients (91.3% had simple MDR‐TB) in South Africa in 2007. In C208 Stage 1, 23 patients were assigned to the bedaquiline cohort to add 8‐week bedaquiline in standard therapy (400 mg once daily for the first 2 weeks and 200 mg three times a week for the remaining 6 weeks), whereas the other 24 patients received placebo for 8 weeks. The study found a quicker sputum culture conversion (hazard ratio, 2.253; 95% confidence interval, 1.08–4.71; *p* = 0.031) and a higher culture conversion rate at week 24 (17/21, 81% vs. 15/23, 65%, *p* = 0.242) in the bedaquiline group compared with the placebo group. In trial C208 Stage 2, bedaquiline administration was prolonged to 24 weeks (400 mg once daily for the first 2 weeks and 200 mg three times a week for the remaining 22 weeks) in 160 subjects from eight countries. C208 Stage 2 substantially improved the culture conversion rate at week 24 (52/66, 79% vs. 38/66, 58%, *p* = 0.008) and the cure rate at week 120 (38/66, 58% vs. 21/66, 32%, *p* = 0.003) compared to the control group with placebo treatments. Actually, it was Trial C208 Stage 2 that underpinned the recommended use of bedaquiline for the later clinical application. The researchers generalized their findings to a larger population of 233 individuals, 37% of whom were infected with complicated MDR‐TB, in a single‐arm trial (Trial C209) (Pym et al., 2016). In this study, the culture conversion rate at week 24 reached 79.5%, which was similar to the results of Trial C208.

**Table 1:** Summary of prospective studies reporting on bedaquiline‐containing treatment for MDR‐TB patients

Corresponding author, countries, publication year	No. of cases in efficacy assessment/no. of cases in the study	No. (%) pre‐XDR‐TB cases	No. (%) XDR‐TB cases	Regimen design	No. (%) of cases taking LZD	No. (%) cases HIV‐infected	The median time to culture conversion (days)	Sputum culture conversion at week 24	Treatment outcome evaluation
Andreas H. Diacon, South Africa, 2012	21/23	2 (8.7%)	0 (0%)	A background regimen plus 8‐week BDQ or placebo.	0 (0%)	3 (13%)	78	17/21 (81%)	11 (51%) had favorable outcome.
Brian Dannemann, multi‐country^a^, 2014	66/80	15 (28%)	0 (0%)	A background regimen plus 24‐week BDQ or placebo.	0 (0%)	5 (8%)	83	52/66 (79%)	38 (58%) had favorable outcome.
Alexander S. Pym1, multi‐country^b^, 2017	205/233	44 (21%)	37 (18%)	A background regimen plus 24‐week BDQ.	34 (14%)	0 (0%)	57	163/205 (79%)	128 (62%) had favorable outcome.
Norbert Ndjeka, South Korea, 2015	41/91	41 (45%)	33 (36%)	An optimized regimen of at least three drugs plus BDQ for 24 weeks.	63 (69%)	54 (59%)	45	33/48 (69%)	Not specified. Only early efficacy assessment.
Keertan Dheda, South Africa, 2018	68/272	0 (0%)	272 (100%)	A DST‐individualized regimen of a median of five effective drugs.	55 (81%)	35 (51%)	Not specified	46/68 (68%)	45 (66%) had favorable outcome.

MDR‐TB = multidrug‐resistant tuberculosis; pre‐XDR‐TB = pre‐extensively drug‐resistant tuberculosis; XDR‐TB = extensively drug‐resistant tuberculosis; LZD = linezolid; BDQ = bedaquiline; DST = Drug susceptibility testing.

a This study enrolled patients from eight countries in Brazil, India, Latvia, Peru, the Philippines, Russia, South Africa, and Thailand.

b This study enrolled patients from 11 countries in China, South Korea, Estonia, Latvia, Turkey, Ukraine, Peru, the Philippines, Russia, South Africa, and Thailand.

Ndjeka and colleagues studied HIV‐infected patients with TB (Ndjeka et al., 2015). A prospective single‐arm study introduced bedaquiline into the background treatment for 91 patients with MDR‐TB with a HIV prevalence of 59%, and 33 of 48 (69%) patients who were culture‐positive before bedaquiline start had culture conversion at week 24.

Clinical research in bedaquiline has witnessed a significant shift in focus from simple MDR‐TB to complicated MDR‐TB cases, especially to XDR‐TB, which is medically challenging to treat. Recently, Dheda et al. reported the results of a prospective cohort study conducted in 272 XDR‐TB individuals (Olayanju et al., 2018), showing that the bedaquiline‐containing regimen resulted in a higher favorable treatment outcome rate than regimens that did not include bedaquiline (66.2% vs. 13.2%). However, the combination with linezolid might have contributed to this considerable difference as more than 80% of patients in the bedaquiline group received linezolid concurrently compared with none of patients in the non‐bedaquiline group.

Taken together, available evidence from six prospective studies suggests a marked improvement in the effectiveness of the treatment regimen after the introduction of bedaquiline with or without linezolid for simple and complicated MDR‐TB.

#### 1.1.2 Treatment benefit of bedaquiline shown in retrospective studies varying in different countries

There have been several retrospective studies describing compassionate use of bedaquiline in many countries (Figure 1; Table 2). A total of four studies from France (Guglielmetti et al., 2014) (Guglielmetti et al., 2017), Germany (Olaru, Heyckendorf, Andres, Kalsdorf, & Lange, 2017), and Belarus (Skrahina et al., 2016) all described the high culture conversion rate at week 24 which exceeded 90%, and one reached 100%, among the patients with highly resistant forms of TB. The data indicated a beneficial effect of the bedaquiline use in patients in European countries. Compared with the satisfactory performance of bedaquiline in Europe, investigations conducted in Asian countries including India (Udwadia, Ganatra, & Mullerpattan, 2017) and South Korea (Kim et al., 2018) showed inconsistent results. Conversion rates at week 24 were between 60 and 70%. One possible explanation was that there were generally more than 90% patients in Europe who received concurrent linezolid, which was much more common than in Asian patients. To our knowledge, the largest retrospective single‐arm study was conducted by Migliori et al. at 25 centers in 15 countries on five continents, enrolling 428 patients with MDR‐TB (Borisov et al., 2017). The results from this wide‐ranging study showed that bedaquiline‐containing regimens achieved treatment success rates of 71.3%, supporting the potential of bedaquiline use in nonexperimental conditions. In this investigation, the treatment success rate among XDR‐TB cases was higher than that of MDR‐TB cases (90/119, 76.9% vs. 86/128, 67.2%), which raised a question about the optimal use profile of bedaquiline.

**Table 2:** Summary of retrospective studies reporting on bedaquiline‐containing treatment for MDR‐TB patients

Corresponding author, countries, publication year	No. of cases in efficacy assessment/no. of cases in the study	No. (%) pre‐XDR‐TB cases	No. (%) XDR‐TB cases	Regimen design	No. (%) of cases taking LZD	No. (%) cases HIV‐infected	The median time to culture conversion (days)	Sputum culture conversion at week 24	Treatment outcome evaluation
Jérôme Robert, France, 2015	29/35	14 (40%)	19 (54%)	A DST‐individualized regimen including a median of five effective drugs. Nine patients took surgical treatment.	33 (94%)	0 (0%)	85	28/29 (97%)	Not specified. Only early efficacy assessment.
Alena Skrahina, Belarus, 2016	197/197	59 (29.9%)	128 (65.0%)	Not specified	Not specified	Not specified	Not specified	186/197 (94.4%)	Not specified. Only early efficacy assessment.
Christoph Lange, Germany, 2017	20/30	9 (30%)	15 (50%)	A DST‐individualized regimen of a median of six effective drugs. BDQ was started within 1 month of treatment start.	Not specified	Not specified	49	20/20 (100%)	Not specified. Only early efficacy assessment.
Lorenzo Guglielmetti, France, 2017	41/45	17 (38%)	24 (53%)	A DST‐individualized regimen of a median of seven effective drugs. Of note, 33 were treated BDQ treatment >190 days.	43 (95.6%)	2 (4.4%)	89	40/41 (97.6%)	36 (80%) had favorable outcome, 5 were lost to follow‐up, 3 died, and acquired BDQ resistance.
Jai. Mullerpattan, India, 2017	17/20	7 (35%)	13 (65%)	A regimen of a median of six drugs.	20 (100%)	1 (5%)	81	11/17 (64.7%)	11 cured, 7 failed, 2 still on treatment.
Yong‐Soo Kwon, South Korea, 2018	36/39	25 (64.1%)	9 (23.1%)	The median duration of BDQ was 323 days.	17 (44%)	Not specified	84	24/36 (66.7%)	Not specified. 5/39 cases discontinued treatment before 6 months.
Giovanni Battista Migliori, multi‐country^a^, 2017	247/428	Not specified	195 (45.6%)	A DST‐individualized regimen.	351 (82%)	94/425 (22.1)	60	Not specified	51/428 discontinued BDQ. Of note, 154/247 (62.4%) cured, 22 completed, 33 died, 18 default, 19 failed.

MDR‐TB = multidrug‐resistant tuberculosis; pre‐XDR‐TB = pre‐extensively drug‐resistant tuberculosis; XDR‐TB = extensively drug‐resistant tuberculosis; LZD = linezolid; BDQ = bedaquiline.

a This study enrolled patients from 15 countries in Africa, Asia, Western and Eastern Europe, Oceania, and Southern America (Argentina, Australia (Victoria State), Belarus, Belgium, Greece, India, Italy, the Netherlands, Peru, Portugal, Russian Federation (Arkhangelsk and Moscow city), South Africa, Spain, Sweden, and United Kingdom).

#### 1.1.3 Concerns over bedaquiline safety and resistance

At the preliminary stage of the research on bedaquiline, its cardiac safety, especially the potential risk of prolonging the QT interval, was the primary concern for physicians. With over a decade of experience, initial observations suggested a relatively favorable safety profile for bedaquiline because its discontinuation occurred in only 0.6% (8/1266) of patients due to QT interval prolongation (Pontali et al., 2017).

Currently, emerging bedaquiline resistance might be an overriding concern that curtails this enthusiasm. There are several issues that require consideration and discussion. First, a reliable definition of bedaquiline resistance has not been established, and the resistance tended to be documented as an increase in bedaquiline minimal inhibitory concentration in previous clinical studies (Veziris et al., 2017). The breakpoint is accepted to be 0.25 mg/L in 7H10/7H11 medium, and the single breakpoint might not completely differentiate the resistant strains from those that are drug‐susceptible (Veziris et al., 2017). Second, cross‐resistance between bedaquiline and clofazimine had been discovered even before bedaquiline was widely used, represented by mutations in the *Rv0678* and *pepQ* genes (Nguyen, Anthony, Bañuls, Vu, & Alffenaar, 2018). This mechanism potentially increased the risk of primary resistance to bedaquiline besides the transmission factor, thus calling for the background assessment of bedaquiline and clofazimine resistance before treatment initiation especially for those who had received clofazimine‐containing regimens. Third, as mentioned above, the long half‐life of bedaquiline would give rise to the monotherapy exposure in the case of patients who have not culture converted when bedaquiline‐containing treatment is discontinued due to treatment nonadherence, which may select for bedaquiline resistance. Therefore, bedaquiline resistance is supposed to be monitored carefully, even after bedaquiline is no longer administrated. Fourth, the fears of acquired resistance to bedaquiline in patients with XDR‐TB or resistance beyond XDR are controversial. Many physicians are apprehensive about introducing bedaquiline into an inadequate background regimen that may result in further transmission of bedaquiline resistance. However, many scholars argued that concerns about resistance should not forestall the use of bedaquiline in patients with XDR‐TB because acquired drug resistance in *M. tuberculosis* mostly results from mutations within a single bacterial chromosome. Thus, the use of bedaquiline in patients with XDR‐TB will not cause the spread of bedaquiline resistance among patients with either simple MDR‐TB or pre‐XDR‐TB. Moreover, the combination of bedaquiline literally offers a rare opportunity for successful treatment of patients with XDR‐TB and prevention of XDR‐TB transmission, and even, if bedaquiline resistance is selected during the treatment, the clinical outcome for these patients would not be worse than those without a bedaquiline‐containing regimen (Kunkel, Furin, & Cohen, 2017). Therefore, perhaps, the greatest challenge confronting physicians is how to use bedaquiline to design an effective regimen that could prevent acquired resistance to bedaquiline. A prerequisite for successful treatment regimen is the early availability of reliable drug‐susceptible testing for the infective strain to all candidate agents. It is crucial to determine the resistance profile (rifampicin‐resistant TB, simple MDR‐TB, pre‐XDR‐TB, or XDR‐TB) before treatment initiation and ensure adequate effective drugs. Recently, bedaquiline has been recommended as one of the Group A drugs in the longer regimen for the treatment of MDR‐TB (WHO, 2018a). It is recommended that bedaquiline should be administered in combination with at least one drug with both bactericidal and sterilizing activity including fluoroquinolones, linezolid, delamanid, or pretomanid (Guglielmetti, Le Dû, Fréchet‐Jachym, & Mitnick, 2016; Veziris et al., 2017) and a total of five effective drugs should be ensured during the initial phase and four effective drugs are needed in the consolidation phase. Adding bedaquiline alone to a failing regimen should be dissuaded.

### 1.2 DELAMANID

Delamanid, a mycolic acid biosynthesis inhibitor, was approved by the European Medicines Agency in 2014 but has not been approved by the FDA. It was estimated that 1,429 patients had received delamanid by March 2018. Compared to bedaquiline, the investigation into the clinical effectiveness of delamanid, while promising, is still in its early stages. The EBA of a 200 mg daily dose was 0.052 over days 0–14 (Diacon et al., 2011), supporting the bactericidal potential of this drug.

#### 1.2.1 Enhancement of culture conversion rate from little high‐quality evidence

There are three prospective studies, including a randomized placebo‐controlled trial (Trial 204), an open‐label cohort study (Trial 208), and a follow‐up study (Trial 116). In Trial 204 (Gler et al., 2012), patients who were assigned to receive delamanid at a dose of 100 mg twice daily and 200 mg twice daily had a higher conversion rate of 45.4% (64/141) and 41.9% (57/136), respectively, at 2 months in comparison with those in the placebo group (29.6%, 37/125). Trial 208 (Skripconoka et al., 2013) extended the administration of delamanid for an additional 6 months among patients who completed Trial 204. Favorable treatment outcome rates among patients receiving delamanid for 8 months, 6 months, 2 months, and placebo reached 74.9%, 74.2%, 53.8%, and 57.5%, respectively, which appeared to conclude that treatment‐containing delamanid for 6 months or more (long‐term) could improve outcomes. However, 25.3% of patients who received delamanid for less than 2 months (short‐term) defaulted during the treatment, compared to 7.8% of patients who received long‐term delamanid. Moreover, improved treatment outcomes were not observed in patients with XDR‐TB who received long‐term delamanid compared with those administered with short‐term delamanid. Therefore, more well‐designed prospective studies are needed to support the evidence of treatment benefit with delamanid.

To date, retrospective studies reporting the effectiveness of delamanid have been descriptive in nature (Table 3), which precluded researchers from looking at some crucial issues, especially the time to sputum culture conversion and the treatment outcome. Cohorts from South Africa (Mohr et al., 2018), Hongkong (Chang et al., 2018), Latvia (Kuksa, Barkane, Hittel, & Gupta, 2017), and South Korea (Kim et al., 2018) focused on complicated MDR‐TB and reported a conversion rate at week 24 of greater than 70%. The first multicenter study conducted in resource‐limited settings also reported an encouraging 24‐week conversion rate of 80%, although 91% of patients were concurrently treated with linezolid (Hafkin, Hittel, Martin, & Gupta, 2017). Nevertheless, another multicenter observational study supported by Médecins Sans Frontières was conducted in seven countries (Armenia, Belarus, Georgia, India, Russia, South Africa, and Swaziland) and showed that 67.6% (25/37) of patients culture converted by 6 months (Hewison et al., 2017). This discrepancy may be due to the high proportion of participants who had previously failed MDR‐TB treatment.

**Table 3:** Summary of retrospective studies reporting on delamanid‐containing treatment for MDR‐TB patients

Corresponding author, countries, publication year	No. of cases in the study	No. (%) pre‐XDR‐TB cases	No. (%) XDR‐TB cases	Regimen design	No. (%) of cases taking LZD	No. (%) of cases taking BDQ	No. (%) cases HIV‐infected	The median time to culture conversion	Sputum culture conversion at week 24	Treatment outcome evaluation
Erika Mohr, South Africa, 2018^a^	103	20 (19.4%)	17 (16.5%)	An individualized regimen with at least five effective drugs.	40–50%	32 (31%)	79 (77%)	Not specified	22/31 (71%)	Among 22 after the end of therapy, seven (32%) had favorable outcome, seven were lost to follow‐up, five died.
Kwok‐Chiu Chang, China, 2018	11	4 (36.4%)	7 (63.6%)	An individualized treatment regimen	100%	0%	0%	Not specified	10/11 (91%)	Nine (82%) cured, one still on treatment, one acquired LZD and DLM resistance.
Norbert Hittel, Germany, 2017^b^	19	8 (42.1%)	9 (47.4%)	An individualized regimen. The mean duration of DLM was 31.2 weeks.	14 (74%)	0%	1 (5.3%)	Not specified	Not specified	16 (84.2%) cured, 3 were lost to follow‐up.
Jeffrey Hafkin, multi‐country^c^, 2017	78	26 (33%)	44 (56%)	An individualized regimen of a mean of 3.3–4.3 active drugs.	60 (91%)	12 (18%)	12 (15%)	Not specified	43/54 (80%)	Not specified
Cathy Hewison, multi‐country^d^, 2017	53	14 (27.5%)	27 (52.9%)	98.1% (52/53) received delamanid for an indication of <4 effective drugs in the regimen	Not specified	Not specified	8 (17%)	Not specified	25/37 (68%)	At 6 months, 39 (73%) had favorable outcome, five failed, two were lost to follow‐up, seven died.
Doosoo Jeon, South Korea, 2018	32	20 (62.5%)	6 (18.7%)	A regimen including a median of five companion drugs	23 (71.9%)	0%	0%	57 days	13/14 (94%)	Not specified
Yong‐Soo Kwon, South Korea, 2018	11	6 (54.5%)	3 (27.2%)	A regimen of a median of five drugs.	6 (54.5)	0%	Not specified	122 days	8/8 (100%)	Not specified. 1/11 cases discontinued treatment before 6 months.

MDR‐TB = multidrug‐resistant tuberculosis; pre‐XDR‐TB = pre‐extensively drug‐resistant tuberculosis; XDR‐TB = extensively drug‐resistant tuberculosis; LZD = linezolid; DLM = delamanid; BDQ = bedaquiline.

a This study also included 21 patients with rifampicin‐mono resistant TB.

b These corresponding authors worked for Ostuka Pharmaceutical Corporation.

c This study enrolled patients from Europe, Asia, and Africa.

d This study enrolled patients from seven countries including Armenia, Belarus, Georgia, India, Russia, South Africa, and Swaziland.

Overall, the net effect of delamanid introduction has barely been evaluated based on the paucity and weakness of the existing evidence. Research on delamanid has been mostly restricted by the absence of a comparative control group. Well‐organized interventional clinical trials are urgently needed to clarify the clinical value of delamanid.

### 1.3 Combination of bedaquiline and delamanid

Since 2016, case reports (Lachâtre et al., 2016; Tadolini et al., 2016) and case series continued to be published on the combination of bedaquiline and delamanid as salvage therapy for individuals with few treatment options. Because of the potential for QT interval prolongation effects of both drugs, the major concern of a regimen containing the combination of bedaquiline and delamanid would still be the theoretical safety profile, and for that reason, the WHO has not yet recommended their combined use. Although no prospective studies have been published, current observational studies showed promising preliminary results. In 2017, three case series reported that of 12 patients treated concomitantly with bedaquiline and delamanid and 5 (41.7%) patients had prolongation of the QT interval corrected with Fridericia's formula (QTcF) of greater than 500 ms, but no arrhythmias were observed (Guglielmetti et al., 2018; Kim et al., 2018; Maryandyshev et al., 2017), compared to 3.2% (42/1301) among patients who received bedaquiline without delamanid (Pontali et al., 2017). In 2018, the largest cohort study showed that no episode of QTcF values greater than 500 ms or cardiac arrhythmias were detected among a total of 28 patients under active monitoring (Ferlazzo et al., 2018). Nevertheless, close electrocardiograph monitoring remains mandatory.

## 2 CONCLUSIONS

Owing to the unmet needs for patients with MDR‐TB, bedaquiline and delamanid are required, with the ultimate aim of eliminating *M. tuberculosis* disease. Based on the current evidence, both bedaquiline and delamanid might offer fresh opportunities for successful treatment management. The improvement of the culture conversion rate and a favorable treatment outcome after the introduction of bedaquiline has been shown by many studies. Prevention of acquired resistance to bedaquiline and optimal use of bedaquiline are extremely challenging and complicated. Research on delamanid remains unsatisfactory, and further prospective studies are required to support clinical applications of these drugs.

## CONFLICT OF INTEREST

We declare no competing interests.
